# Correlation Between Hippocampus MRI Radiomic Features and Resting-State Intrahippocampal Functional Connectivity in Alzheimer’s Disease

**DOI:** 10.3389/fnins.2019.00435

**Published:** 2019-05-07

**Authors:** Qi Feng, Mei Wang, Qiaowei Song, Zhengwang Wu, Hongyang Jiang, Peipei Pang, Zhengluan Liao, Enyan Yu, Zhongxiang Ding

**Affiliations:** ^1^Department of Radiology, Affiliated Hangzhou First People’s Hospital, Zhejiang University School of Medicine, Hangzhou, China; ^2^Department of Radiology, Zhejiang Provincial People’s Hospital, Hangzhou Medical College, Hangzhou, China; ^3^Department of Radiology and BRIC, The University of North Carolina at Chapel Hill, Chapel Hill, NC, United States; ^4^GE Healthcare Life Sciences, Hangzhou, China; ^5^Department of Psychiatry, Zhejiang Provincial People’s Hospital, Hangzhou Medical College, Hangzhou, China

**Keywords:** Alzheimer’s disease, resting-state functional magnetic resonance imaging, functional connectivity, hippocampus, radiomics

## Abstract

Alzheimer’s disease (AD) is a neurodegenerative disease with main symptoms of chronic primary memory loss and cognitive impairment. The study aim was to investigate the correlation between intrahippocampal functional connectivity (FC) and MRI radiomic features in AD. A total of 67 AD patients and 44 normal controls (NCs) were enrolled in this study. Using the seed-based method of resting-state functional MRI (rs-fMRI), the whole-brain FC with bilateral hippocampus as seed was performed, and the FC values were extracted from the bilateral hippocampus. We observed that AD patients demonstrated disruptive FC in some brain regions in the left hippocampal functional network, including right gyrus rectus, right anterior cingulate and paracingulate gyri, bilateral precuneus, bilateral angular gyrus, and bilateral middle occipital gyrus. In addition, decreased FC was detected in some brain regions in the right hippocampal functional network, including bilateral anterior cingulate and paracingulate gyri, right dorsolateral superior frontal gyrus, and right precentral gyrus. Bilateral hippocampal radiomics features were calculated and selected using the A.K. software. Finally, Pearson’s correlation analyses were conducted between these selected features and the bilateral hippocampal FC values. The results suggested that two gray level run-length matrix (RLM) radiomic features and one gray level co-occurrence matrix (GLCM) radiomic feature weakly associated with FC values in the left hippocampus. However, there were no significant correlations between radiomic features and FC values in the right hippocampus. These findings present that the AD group showed abnormalities in the bilateral hippocampal functional network. This is a prospective study that revealed the weak correlation between the MRI radiomic features and the intrahippocampal FC in AD patients.

## Introduction

Alzheimer’s disease (AD) is a neurodegenerative disease with main symptoms of chronic primary memory loss and cognitive impairment. AD has become a public health problem due to its hidden onset, high incidence, and lack of effective drug treatment. The exact cause of AD is still unclear. It is believed some may be related to heredity, neurotransmitter changes, virus infection, immune dysfunction, and free radical damage. Nowadays, the clinical diagnosis of AD is based on cognitive measures and one or more biomarkers including structural MRI, PET, and cerebrospinal fluid analysis of amyloid β or tau proteins (Bruno et al., 2007). Researchers are investigating non-invasive neuroimaging biomarkers for the early diagnosis of AD (Claudia et al., 2010; Yong et al., 2014).

Cell degeneration in the hippocampus plays an important role in the onset of AD. It proved to be the cause of AD that amyloid-β plaques and Tau proteins are selectively deposited in the special cortex of the hippocampus in AD patients (Ball, 1997; Guzman et al., 2013). These special cortexes are the main pathways that connect the hippocampus to the other cortexes of the brain. A large number of magnetic resonance imaging (MRI) studies have revealed atrophy and some other changes in microstructure in the hippocampus in AD dementia (De et al., 2015;Mak et al., 2017).

MRI is an important technique for radiomics. MR images can acquire numerous sequences and do not receive a radiation dose. MRI shows both the structural change and the functional dynamic change. Several MRI techniques have been applied in AD research, including voxel-based morphometry (Whitwell et al., 2007), diffusion tensor imaging (DTI) (Denise et al., 2004), resting-state functional MRI (rs-fMRI; Wang et al., 2011), as well as radiomics analysis (Qi et al., 2018). Radiomics is a new frontier subject based on quantitative imaging, feature calculation, feature selection, and model construction. It uses a large number of automated feature extraction algorithms to transform the original image data into first-order or higher-order data, and then analyzes the deep relationship between the data to further improve the accuracy of clinical diagnosis and prognostic value. Radiomics has strong power for radiotherapy, chemotherapy, and immunotherapy evaluation (Horvat et al., 2018; Sun et al., 2018; Wang et al., 2018), cancer patients survival prediction (Kickingereder et al., 2016), molecular subtyping of tumor (Lu et al., 2018), and cancer recurrence prediction (Li H. et al., 2017). Nowadays, radiomics is also applied to non-tumor diseases, such as attention deficit hyperactivity disorder (Port, 2018) and autism spectrum disorder (Chaddad et al., 2017a). In this context, we select the high-resolution T1-weighted MR images for hippocampal microstructural radiomics analysis.

**Table 1:** Demographics and cognitive characteristics of the participants.

	AD patients	NC group	Statistic	*P-*value
Sample size	67	44	NA	NA
Age (years, mean ± SD)	68.75 ± 11.69	65.48 ± 9.69	1.54	0.13
Gender (male/female)	29:38	20:24	0.05^∗^	0.82^∗^
Education (years, mean ± SD)	6.10 ± 3.78	7.11 ± 3.36	−1.44	0.15
MMSE	17.16 ± 5.54	29.09 ± 0.77	−14.17	<0.01

*SD, standard deviation; MMSE, Mini-Mental State Examination. AD, Alzheimer’s disease; NC, normal control. Statistics were calculated with t-tests, unless otherwise indicated. ^∗^x^2^-test was used.*

Rs-fMRI is a functional MRI technique that based on blood oxygen level dependent (BOLD), and has emerged as one of the most important techniques for analysis of human brain function. Functional connectivity (FC) is the indirect reflection of synaptic connections. Rs-fMRI FC analysis is increasingly used to detect brain network changes in AD. There are two main methods used in rs-fMRI FC analysis. One is the “seed-based” approach, and the other is independent component analysis (ICA). In seed-based analysis, first, you need to set up a region of interest (ROI), then select BOLD signal fluctuations from the ROI, and associate them with BOLD signal fluctuations for all other voxels in the brain (Sheline and Raichle, 2013). The default mode network (DMN) is most commonly shown to be active when a person is not focused on the outside world and the brain is at wakeful rest, such as during daydreaming and mind-wandering. It is highly correlated with cognitive function (Menon, 2011). FC altered in DMN becomes a potential non-invasive biomarker in diagnosis of AD and amnestic mild cognitive impairment (aMCI) (Damoiseaux et al., 2012; Toussaint et al., 2014; Yu et al., 2016; Alderson et al., 2017). Functional activity in the hippocampus is the hot topic of AD research. One study found decreased synchrony of low-frequency fluctuations within the hippocampus in AD patients (Shi-Jiang et al., 2002). An ICA study of fMRI showed that the hippocampal activity diminished in AD (Wu et al., 2011). Intrinsic connectivity altered in hippocampal functional networks has been observed in aMCI patients (De et al., 2017). However, most of the rs-fMRI studies were focused on the DMN and some local brain regions; little is known regarding the hippocampus functional network.

**Table 2:** Regions showing altered FC for AD patients and NC subjects with the left hippocampus as seed.

Anatomical region	BA	MNI coordinates (*x*, *y*, *z*)	Cluster size (voxels)	*T*-value
REC.R, ACG.R, ORBmid.R, CAU.R	11/10/32/25	−6, 39, −15	344	−3.458
PCUN.L, PCUN.R, DCG.L	31/7	−15, −48, 33	264	−3.762
ANG.R, MOG.R	39/19/7	39, −66, 39	280	−3.600
ANG.L, MOG.L, IPL.L	39/40/19	−33, −60, 33	337	−3.492

*BA, Brodmann’s area; x, y, z, coordinates of peak locations; T-value, t statistical values of peak locations; L, left; R, right; REC, gyrus rectus; ACG, anterior cingulate and paracingulate gyri; ORBmid, middle frontal gyrus, orbital part; CAU, caudate nucleus; PCUN, precuneus; DCG, median cingulate and paracingulate gyri; ANG, angular gyrus; MOG, middle occipital gyrus; IPL, inferior parietal but supramarginal and angular gyri.*

Many studies investigated the altered structure or function in the hippocampus in AD patients. Nevertheless, little is known regarding the relationship between hippocampal microstructure and FC in AD. The hypothesis of the present study is that the local structural change in hippocampus is correlated to the functional changes of the brain in AD patients. The aim of the study was to investigate the correlation between hippocampal MRI radiomic features and intrahippocampal FC in AD, and to explore non-invasive imaging biomarkers for early diagnosis of AD.

## Materials and Methods

### Study Cohort

There were 82 AD patients and 50 normal controls (NCs) recruited initially. AD patients were enrolled prospectively in the study at the Zhejiang Provincial People’s Hospital from September 2016 to June 2018. The NCs were volunteers collected from the health promotion center of the hospital. All subjects were right-handed and provided written informed consent. This study was approved by the Ethics Committee of Zhejiang Provincial People’s Hospital (No. 2012KY002) and had been carried out in accordance with the Declaration of Helsinki.

**Table 3:** Regions showing altered FC for AD patients and NC subjects with the right hippocampus as seed.

Anatomical region	BA	MNI coordinates (*x*, *y*, *z*)	Cluster size (voxels)	*T*-value
ACG.L, ACG.R, DCG.R	24/32/23	−9, −21, 27	429	−4.703
SFGdor.R, PreCG.R, MFG.R	6/8	18, 6, 51	276	−3.793

*BA, Brodmann’s area; x, y, z, coordinates of peak locations; T-value, t statistical values of peak locations; L, left; R, right; ACG, anterior cingulate and paracingulate gyri; DCG, median cingulate and paracingulate gyri; SFGdor, superior frontal gyrus, dorsolateral; PreCG, precentral gyrus; MFG, middle frontal gyrus.*

All subjects underwent medical history collection, laboratory examination, neuropsychological test, physical examination, and conventional brain MRI scans. The neuropsychological tests included the Mini-Mental State Examination (MMSE) and Montreal Cognitive Scale (MoCA). AD patients were diagnosed in terms of the revised NINCDS-ADRDA (National Institute of Neurological and Communicative Disorders and Stroke and the Alzheimer’s Disease and Related Disorders Association) criteria (Amp, 2011) with MMSE score ≤ 24 and MoCA score ≤ 26. The inclusion criteria for NC subjects were as follows: (1) no neurological deficits, such as hearing or vision loss; (2) no neurological or mental diseases, such as stroke, epilepsy, or depression; (3) no evidence of infarction, hemorrhage, or tumor on routine MRI; and (4) MMSE score ≥ 28. For both AD and NC groups, the exclusion criteria were (1) stroke; (2) brain trauma; (3) brain tumors, Parkinson’s disease, epilepsy, and other neurological diseases that cause memory disorders; (4) serious anemia, hypertension, diabetes, and other systemic diseases; (5) history of mental illness; and (6) signal abnormalities in the medial temporal lobe caused by infectious or vascular factors on MRI FLAIR or T2 images (Dubois et al., 2007). There were 73 AD patients and 45 NC subjects who completed all the required MR sequences successfully.

### MRI Acquisition

Each subject underwent both structural and fMRI examinations on a 3.0-T MR scanner (Discovery MR750; GE Healthcare, Waukesha, WI, United States). The structural MRIs were acquired using a high-resolution three-dimensional T1-weighted magnetization-prepared rapid gradient echo (MPRAGE) sagittal sequence with scanning parameters of repetition time (TR) = 6.7 ms, echo time (TE) = 2.9 ms, inversion time (TI) = 450 ms, slice thickness/gap = 1/0 mm, FOV = 256 × 256 mm^2^, flip angle = 12°, matrix = 256 × 256; there were 192 sagittal slices collected from each subject. The rs-fMRI images were acquired using an echo-planar imaging (EPI) sequence with scanning parameters of TR = 2,000 ms, TE = 30 ms, slice thickness/gap = 3.2/0 mm, FOV = 220 × 220 mm^2^, and flip angle = 90°. Each rs-fMRI sequence contained 210 time points and each time point contained 44 slices. During rs-fMRI, all subjects were instructed to keep still and keep their eyes closed, but to not fall asleep.

### Preprocessing of Resting-State Functional MRI

The image analysis of rs-fMRI after acquisition consists of preprocessing and FC analysis. The preprocessing was performed using the Data Processing Assistant for rs-fMRI (DPARSF^1^). The preprocessing steps were as follows:

(1) removing the first 10 time points (considering the time when the magnetic field of the machine was stable and the time when the subject adapted to the environment);
(2) slice timing correction and head motion correction;
(3) normalization to the Montreal Neurological Institute (MNI) space and resampling (with voxels of 3 × 3 × 3 mm);
(4) spatial smoothing using a 4-mm isotropic Gaussian kernel; removing the linear trend; bandpass filtering (0.01–0.08 Hz); and
(5) regression of covariates, including the six head motion parameters, the white matter, and cerebrospinal fluid signal.

### Segmentation of Hippocampus

The 3D T1-weighted MPRAGE images were used in the hippocampus segmentation using an efficient learning-based deformable model (Wu et al., 2017). A joint classification and regression model was established to predict the location of the hippocampus. In the training stage, the extracted features were used to train the structured random forest classifier, and in the testing stage, the extracted features were input into the classifier in order to predict the segmentation of each hippocampus, and the prediction segmentation is iteratively improved through the training model. Finally, the hippocampal shape model gradually deformed to the target image to adapt the target hippocampus. We used this method to segment the right and left hippocampus, respectively. The segmentation results had been checked by a senior neuroradiologist, and the data with poor segmentation quality were resegmented.

### Resting-State Functional Connectivity Analysis

The FC analysis of the bilateral hippocampus network was performed using REST plus V1.2^2^. We took the averaged time courses of all voxels in ROI (above segmented left and right hippocampus as defined) as the reference sequence. And Pearson’s correlation was conducted between the BOLD time course within the ROI and each voxel in the brain. Then, ROI-wise whole-brain FC maps were obtained. Fisher’s *r*-to-*z* transformation was used to convert FC maps into normalized zFC maps for subsequent statistical analyses. One-sample *t*-test was conducted in the AD and NC groups to make a mask using Gaussian random field (GFR) correction. Two-sample *t*-test was then performed using the above mask between the AD and NC groups. Then, GFR correction was performed (voxel-level *P* < 0.05, cluster-level *P* < 0.05, two-tailed). Finally, the brain areas of significant differences were obtained. Furthermore, in order to quantify the FC values of the bilateral hippocampus in the brain network for next correlation analysis, the mean *Z*-value within the bilateral hippocampus was calculated using the above segmented left and right hippocampus images as ROI definition. The mean *Z*-value represents the average FC value within the hippocampus. These steps were performed in the left and right hippocampus data, respectively.

### MRI Radiomic Analysis

Artificial Intelligence Kit (A.K) is a commercially available software developed by GE Healthcare Institute of Precision Medicine. It performs data loading, segmentation, feature calculation, feature selection, and model establishment of radiomics.

First, we loaded the original 3D T1-weighted images and ROI images into the A.K. software. Then, image features were calculated including Formfactor, Histogram, Haralick, gray level co-occurrence matrix (GLCM), and gray level run-length matrix (RLM). Formfactor features use mathematical methods to characterize the shape of the lesion, and describe the shape and compactness of the lesion. Histogram features calculate the gray intensity information of the lesion and describe the overall distribution of gray level information. GLCM obtains the co-occurrence matrix by counting the probability of the occurrence of pixel pairs in different directions and displacement vectors. It describes the complexity of the lesion, the level variation, and the degree of texture thickness (Seongjin et al., 2011). Haralick also based on the co-occurrence matrix to extract the corresponding features, but it has directional invariance. RLM obtains the length matrix by calculating the probability of the pixels appearing repeatedly in succession with different directions and displacement vectors. It also describes the complexity of the lesion, the level variation and the degree of texture thickness (Galloway, 1975). We chose “1, 4, and 7” for displacement vectors.

After feature calculation, we added labels “0” and “1” for each subject data representing NC and AD. The preprocessing for feature selection included replaced the abnormal value with the mean; set the training set proportion and testing set proportion to 0.7 and 0.3, respectively, and eliminate the unit restriction for each feature column through normalization. The feature selection steps were as follows. Step 1: *T*-test and rank sum test were used to identify the features with significant differences between the two groups (*P* < 0.05). Step 2: The correlation analysis was used to reduce the dimension. The filter threshold was set to 0.9, and the Spearman rank correlation coefficient was selected. Correlation analysis was performed between any two feature columns; one of the two highly correlated features was removed when the correlation coefficient was greater than 0.9. Step 3: The least absolute shrinkage and selection operator (LASSO) regression model was applied to reduce the dimension using 10-fold cross-validation. This method is applicable to the regression analysis of high-dimensional data. We performed feature selection on the left and right hippocampus data, respectively.

### Statistical Analysis

Demographic, neuropsychological comparison, RS FC comparison of the two groups, and correlation analysis between the bilateral hippocampus radiomic features and FC were performed using SPSS version 22.0. All statistical methods about radiomic analysis were performed using the A.K. software.

## Results

### Comparison of Demographic and Neuropsychological Performance

Among the remaining 73 AD patients and 45 NC subjects, 67 AD patients and 44 NC subjects were finally collected for analysis, who had head motion <3.0 mm translation and 3.0° rotation in any direction during preprocessing of rs-fMRI. Table 1 showed the statistical analysis results of demographics and neuropsychological performance. There was no significant difference between AD and NC subjects in demographics (*P* > 0.05). However, there were statistically significant differences in MMSE performance between the two groups (*P* < 0.05).

### Functional Connectivity Analysis

**FIGURE 1:**
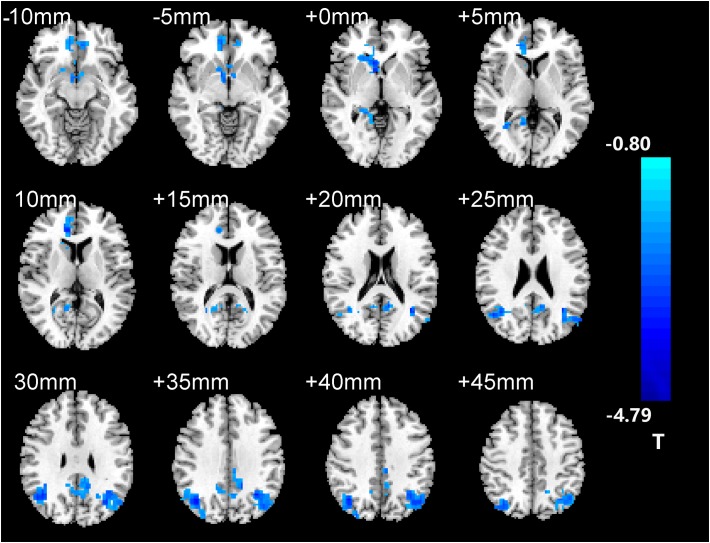
The left hippocampus seed-based functional connectivity (FC) maps in the Alzheimer’s disease (AD) patients compared with normal control (NC) subjects.

**FIGURE 2:**
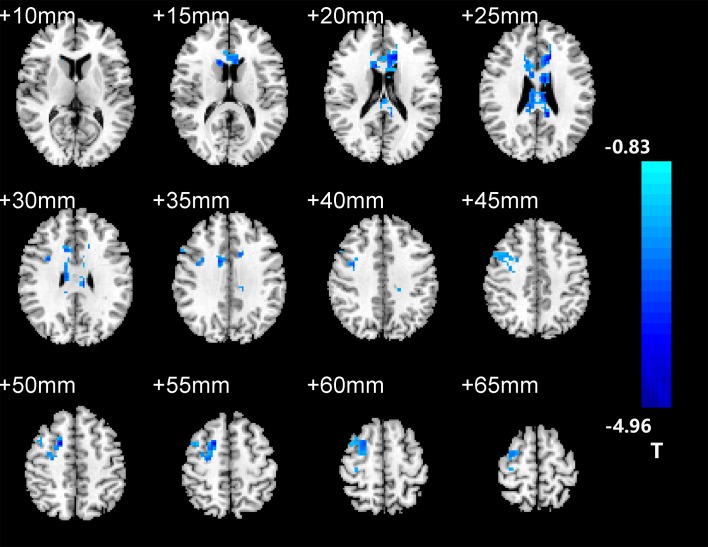
The right hippocampus seed-based FC maps in the AD patients compared with NC subjects.

After two-sample *t*-tests, RS FC had no significant difference between AD and NC within the left hippocampal mask (*t* = 0.34, *P* > 0.05) and the right hippocampal mask (*t* = −1.01, *P* > 0.05). In addition, we observed that FC in some brain regions was disrupted in the left hippocampal functional network in the AD patient group; these regions are right gyrus rectus, right anterior cingulate and paracingulate gyri, right orbital part of middle frontal gyrus, right caudate nucleus, left and right precuneus, left median cingulate and paracingulate gyri, left and right angular gyrus, left and right middle occipital gyrus, and inferior parietal but supramarginal and angular gyri (Figure 1 and Table 2). We also observed some brain regions with decreased FC in the right hippocampal functional network, including left and right anterior cingulate and paracingulate gyri, right median cingulate and paracingulate gyri, right dorsolateral superior frontal gyrus, right precentral gyrus, and right middle frontal gyrus (Figure 2 and Table 3).

**Table 4:** Correlation between the left hippocampal radiomic features and functional connectivity values.

	Type of parameters	r, AD patients (*n* = 67)
RunLengthNonuniformity_angle45_offset1	RLM	−0.261^∗^
LowGreyLevelRunEmphasis_AllDirection_offset1	RLM	0.222
LowGreyLevelRunEmphasis_AllDirection_offset7_SD	RLM	−0.008
Correlation_angle135_offset1	GLCM	−0.260^∗^
GreyLevelNonuniformity_AllDirection_offset1	RLM	−0.282^∗^

*Pearson correlation analyses were conducted to calculate the correlations between these selected features and the left hippocampal FC values. ^∗^P < 0.05. RLM, run-length matrix; GLCM, gray level co-occurrence matrix.*

**Table 5:** Correlation between the right hippocampal radiomic features and functional connectivity values.

	Type of parameters	r, AD patients (*n* = 67)
ShortRunEmphasis_angle90_offset7	RLM	0.203
GLCMEntropy_AllDirection_offset1_SD	GLCM	0.104
SurfaceVolumeRatio	Formfactor	0.174
GreyLevelNonuniformity_AllDirection_offset1	RLM	−0.044

*Pearson correlation analyses were conducted to calculate the correlations between these selected features and the right hippocampal FC values.*

### Radiomic Analysis

**FIGURE 3:**
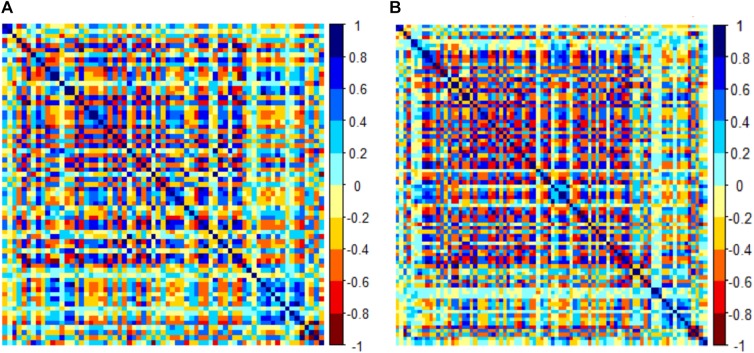
Correlation analysis graph of the left hippocampus **(A)**. Correlation analysis graph of the right hippocampus **(B)**. Each grid represents the feature that enters the correlation analysis after the first step of dimensionality reduction.

**FIGURE 4:**
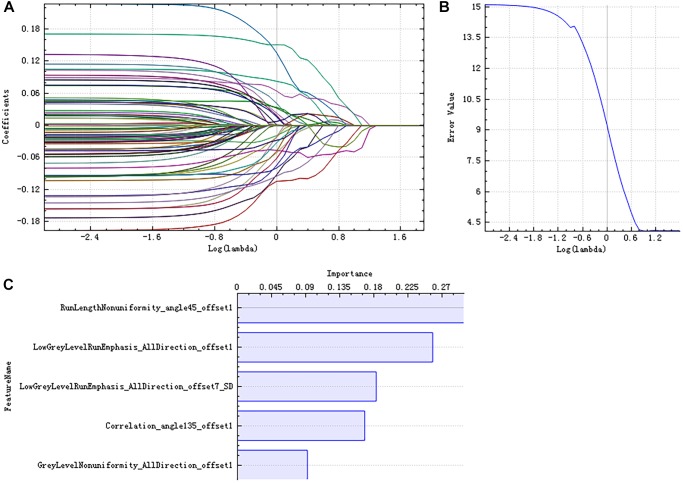
Least absolute shrinkage and selection operator (LASSO) dimensionality reduction of the left hippocampus. Error–lambda graph **(A)**; coefficients–lambda graph **(B)**. We chose λ according to the lowest error rate. Importance of the selected features **(C)**.

**FIGURE 5:**
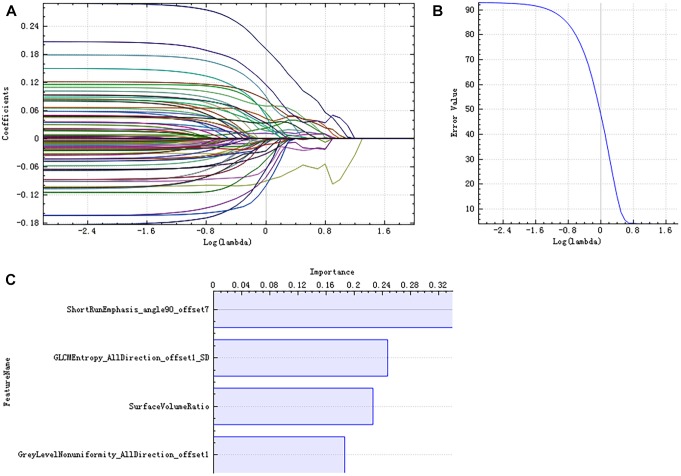
LASSO dimensionality reduction of the right hippocampus. Error–lambda graph **(A)**; coefficients–lambda graph **(B)**. We chose λ according to the lowest error rate. Importance of the selected features **(C)**.

There were 385 features extracted in the bilateral hippocampus after feature calculation. For left and right hippocampus, after *T*-test and rank sum test, the remaining feature numbers were 196 and 215. After correlation analysis, the remaining feature numbers were reduced to 70 and 81 (Figure 3A,B). Finally, using the LASSO regression model, five and four features were selected (Figure 4A–C, 5A–C).

### Correlations Between Radiomic Features and Functional Connectivity

**FIGURE 6:**
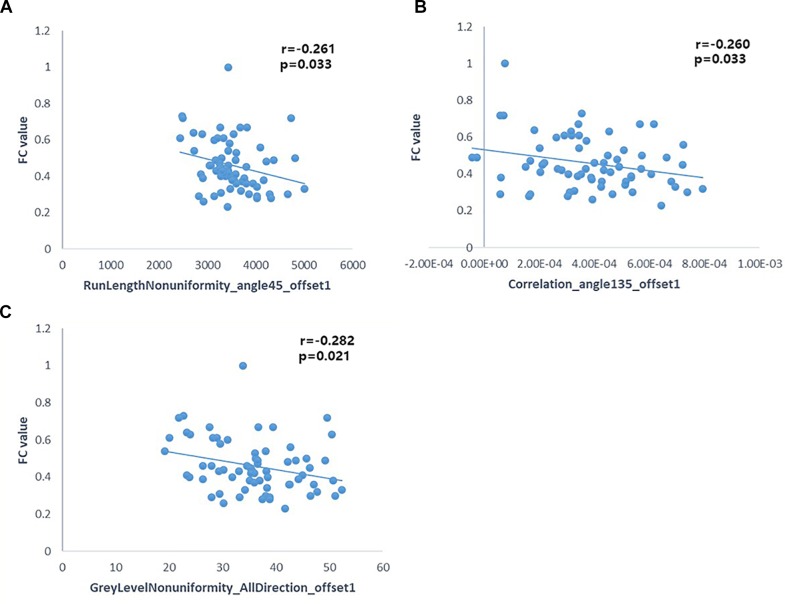
Pearson correlation analysis between some radiomic features and FC of the left hippocampus (*p* < 0.05). Pearson’s correlation analysis between “RunLengthNonuniformity_angle45_offset1” and FC value **(A)**; Pearson’s correlation analysis between “Correlation_angle135_offset1” and FC value **(B)**; Pearson’s correlation analysis between “GreyLevelNonuniformity_AllDirection_offset1” and FC value **(C)**.

Pearson’s correlation analysis suggested that there were three radiomic features (RunLengthNonuniformity_angle45_offset1, Correlation_angle135_offset1, GreyLevelNonuniformity_AllDirection_offset1) associated with FC values in the left hippocampus (*P* < 0.05); the correlation coefficient values were −0.261, −0.260, and −0.282, respectively (Figure 6 and Table 4). However, there were no significant correlations between selected radiomic features and FC values in the right hippocampus (Table 5).

## Discussion

Our study indicated that the AD group showed abnormalities in the left and right hippocampal functional network compared with the NC group. Meanwhile, the present study selected the closely related radiomic features of the bilateral hippocampus. Pearson correlation analysis suggested weak relationship between some radiomic features and FC values in the left hippocampus. It’s a prospective paper to study the correlation between the hippocampal radiomic features and fMRI characteristics in AD.

In addition to hippocampal structural studies, some rs-fMRI studies suggest that hippocampal functional characteristics changed in the AD or aMCI stage. For example, Sorg et al. (2007) found that only selected brain areas such as the hippocampus showed reduced activities in MCI patients. Some AD and MCI studies indicated the destruction of functional connections between the hippocampus and the PCC, medial prefrontal cortex, inferior parietal lobule, and other brain areas (Wang et al., 2006a; Dennis and Thompson, 2014; Krajcovicova et al., 2014). One study showed alterations of three hippocampal subfield functional networks in aMCI patients (De et al., 2017). Our results have demonstrated diminished FC in the right gyrus rectus, right anterior cingulate and paracingulate gyri, bilateral precuneus, bilateral angular gyrus, and bilateral middle occipital gyrus of the left hippocampus functional networks. We also observed some brain regions of decreased FC in the right hippocampal functional network, including bilateral anterior cingulate and paracingulate gyri, right dorsolateral superior frontal gyrus, and right precentral gyrus. Some of these brain regions are major components of the DMN. Most seed-based and ICA studies have shown that functional connections of DMN and other brain networks are reduced in AD or MCI (Greicius et al., 2004; Christian et al., 2007). We observed no increase or decrease of FC in PCC; maybe there are both disruption and compensation effects in the AD stage. However, many studies have reported areas of aberrant increased connectivity in AD (Damoiseaux et al., 2012; Simona et al., 2015; Li M. et al., 2017). An ICA study by Damoiseaux et al. (2012) showed that the FC of posterior DMN began to decrease in the early stage of AD patients, while the connection increased within the anterior and ventral DMN. As the disease progressed, all Internet connections decreased. We observed most of the regions of decreased FC in the bilateral hippocampal functional networks, probably because patients with severe AD were in the majority of our AD subjects. Moreover, the disruption patterns of the left and right hippocampal networks are different in AD and aMCI patients (Wang et al., 2006b; Xie et al., 2013). Our result was consistent with these recent discoveries.

Radiomics analysis has been applied to some neuropsychiatric diseases. A radiomics study about autism spectrum disorder found significant differences in the texture features in the right hippocampus, corpus callosum, cerebellar white matter, and left choroid plexus between patients and controls (Chaddad et al., 2017b). Some texture analysis studies have found that there are texture differences in hippocampus, corpus callosum, and thalamus between AD patients and NCs (Li et al., 2010; Oliveira et al., 2011). The nine radiomic features selected in this study reflect the differences in image gray value distribution, texture characteristics, spatial heterogeneity, and other microstructural information in AD patients. Among the five selected features of the left hippocampus, “RunLengthNonuniformity_angle45_ offset1,” “LowGreyLevelRunEmphasis_AllDirection_offset1,” “LowGreyLevel RunEmphasis_AllDirection_offset7_SD,” and “GreyLevelNonuniformity_AllDirection_offset1” are RLM parameters. The higher the value of “RunLengthNonuniformity” or “GreyLevelNonuniformity” is, the more heterogeneous the lesion is. “LowGreyLevelRunEmphasis” describes the overall brightness of the lesion; the higher the value, the darker the lesion. “Correlation_angle135_offset1” is one of the GLCM parameters. “Correlation” describes the similarity of the gray levels in adjacent pixels and displays the correlation between a pixel and its neighbors across the image. Among the four selected features of the right hippocampus, “ShortRunEmphasis_angle90_offset7” and “GreyLevelNonuniformity_AllDirection_offset1” are RLM parameters. “ShortRunEmphasis” describes the degree of difference in gray value between adjacent pixels of the lesion; the higher the value is, the more complex and heterogeneous the lesion is. “GLCMEntropy_AllDirection_offset1_SD” is a GLCM parameter. “Entropy” describes the complexity of the co-occurrence matrix; the larger the value is, the more complex the co-occurrence matrix will be. “SurfaceVolumeRatio” is a Formfactor parameter; it describes the three-dimensional size and shape of the hippocampus. If the edge irregularity of three-dimensional lesions is large, the ratio is also large, indicating greater heterogeneity. These features extracted from the hippocampus structure reflect high-order imaging patterns and heterogeneity characteristics of microstructure in hippocampus in AD patients.

Although there was no significant difference in hippocampal FC between the two groups, there were significant differences in hippocampal radiomic features. In addition, our study showed weak and negative correlation between the intrahippocampal FC and the radiomic features. It suggests that the changes of hippocampal microstructure appeared before the changes of hippocampal function in AD patients. MR volumetry and DTI studies indicated the decreased volumes and increased mean diffusivity of the hippocampus in AD patients (Palesi et al., 2011; Brueggen et al., 2015). Structural MRI studies have shown that microstructural abnormalities of the hippocampus can be the neuroimaging biomarkers of early cognitive impairment. In future studies, we will pay more attention to the study of hippocampal microstructure to provide imaging basis for the early diagnosis of AD. The occurrence and development of AD is a complex process, and we can obtain more structural and functional information by using a variety of MRI techniques in future studies.

However, there were several limitations in our study. Firstly, a complete 1:1 match in age and sex ratio had not been achieved. Secondly, although patients with MCI were excluded, the severity of the disease in AD patients was not distinguished in the study, and these subjects were not followed up. Mild, moderate, and severe AD patients were all included in the AD group. Lastly, we did not correct the between-group comparison of RS FC adjusting for hippocampal volume. It may influence the results to a certain degree.

In summary, this study observed that there are decreased activity in hippocampus functional network in AD patients. It also indicates that the closely related hippocampal radiomic features can be neuroimaging biomarkers for the diagnosis of AD. Moreover, we explored the correlations between the MRI radiomic features and intrahippocampal FC in AD patients. It provides a very important reference for further understanding the pathogenesis of AD.

## Ethics Statement

This study was carried out in accordance with the recommendations of “Ethics Committee of Zhejiang Provincial People’s Hospital (No. 2012KY002)” with written informed consent from all subjects. All subjects gave written informed consent in accordance with the Declaration of Helsinki. The protocol was approved by the “Ethics Committee of Zhejiang Provincial People’s Hospital.”

## Author Contributions

ZD, EY, and QF designed the experiments. QF, MW, QS, and HJ performed the experiments and analyzed the data. ZW segmented the MR images. ZD, QF, PP, and ZL interpreted the results and drafted the manuscript. All the authors read and approved the final version of the manuscript.

## Conflict of Interest Statement

The authors declare that the research was conducted in the absence of any commercial or financial relationships that could be construed as a potential conflict of interest.

## Footnotes

1 http://rfmri.org/DPARSF

2 http://restfmri.net/forum/RESTplusV1.2
